# Tuberculosis screening among cough suppressant buyers in pharmacies and drug outlets in Guinea: a cross-sectional study

**DOI:** 10.1136/bmjresp-2024-002334

**Published:** 2024-12-31

**Authors:** Aboubacar Sidiki Magassouba, Almamy Amara Toure, Boubacar Djelo Diallo, Gnoume Camara, Desire Lucien Dahourou, Aly Badara Nabe, Souleymane Camara, Adama Marie Bangoura, Hugues Asken Traore, Jonathon R Campbell, Vanessa Veronese, Corinne Simone Collette Merle

**Affiliations:** 1 Republic of Guinea Ministry of Health, Conakry, Guinea; 2 Gamal Abdel Nasser University of Conakry, Conakry, Guinea; 3 National Tuberculosis Program, Conakry, Guinea; 4 Centre National de Formation et de Recherche en Santé Rurale de Maférinyah, Maferinyah, Guinea; 5 Institut de Recherche en Sciences de la Santé, Ouagadougou, Burkina Faso; 6 World Health Organization Special Programme for Research and Training in Tropical Diseases, Geneva, Switzerland; 7 McGill University, Montreal, Quebec, Canada; 8 World Health Organization, Geneva, Switzerland

**Keywords:** Tuberculosis

## Abstract

**Background:**

Tuberculosis (TB) poses a significant public health challenge in Guinea, with an estimated 22 000 TB cases in 2020; an estimated 6125 (28%) cases went undetected. We evaluated an intensified TB case finding strategy in Guinea which targeted customers who bought cough suppressants from pharmacies or drug outlets.

**Methods:**

We involved 25 pharmacies and 25 drug outlets in Matoto, Conakry, Guinea. Pharmacists or outlet owners identified and referred all customers with TB symptoms who were purchasing cough suppressants to healthcare workers for sputum collection either at the pharmacy or drug outlet or at a nearby TB diagnosis and treatment centre (CDT); sputum was subjected to bacteriological testing with acid fast bacilli smear or Xpert MTB/RIF. We assessed factors associated with eventual TB diagnosis using logistic regression and time to TB diagnosis using cox regression and used microcosting to estimate the cost of the intervention in 2020 US$.

**Results:**

From November 2019 to June 2020, we screened 916 people referred from pharmacies or drug outlets with TB symptoms, with median age of 31 years (54% male). Overall, 126 (14%) had bacteriologically confirmed TB. Odds of TB diagnosis were significantly lower with increasing age (adjusted OR (aOR) per additional year=0.98; 95% CI 0.97 to 0.99) and higher among males (aOR=1.57; 95% CI 1.04 to 2.39) and those with symptoms. Those identified at drug outlets had significantly faster time to presentation from symptom onset than pharmacies (adjusted HR=1.73; 95% CI 1.50 to 1.99). The total cost of the intervention per person referred was US$32.66 and per person diagnosed and treated for TB disease of US$237.45.

**Conclusion:**

Intensified TB case finding through pharmacies and drug outlets is a feasible and effective way to increase TB detection in settings where self-medicating is common, and TB is under-detected.

**WHAT IS ALREADY KNOWN ON THIS TOPIC**

- Enhanced, intensified and active tuberculosis (TB) case finding activities are recommended by the WHO for groups where the prevalence of TB is high.
- A large proportion of people with symptomatic TB first access care in the private sector, including seeking medications through pharmacies.

**WHAT THIS STUDY ADDS**

- In Matoto, Conakry, Guinea, a high TB incidence setting, nearly 1 in 7 symptomatic people purchasing cough suppressants at pharmacies or informal drug outlets, had smear positive TB.
- People who visited informal drug outlets did so significantly sooner after symptom onset than did people who visited pharmacies.

**HOW THIS STUDY MIGHT AFFECT RESEARCH, PRACTICE OR POLICY**

- Engaging pharmacies and informal drug outlets in the referral of people for TB disease screening should be considered in high incidence settings.

## Background

 Tuberculosis (TB) remains a significant health problem in Guinea despite enormous efforts that have been made since the creation of the national tuberculosis control program (NTP) in 1990. In 2023, 20 294 cases of TB, all forms included, were detected, of which 16 032 were bacteriologically confirmed with a notification rate of 156 per 100 000 inhabitants.^1^ However, although increasing each year, these reported cases remain well below WHO estimates, suggesting that a substantial number of estimated TB cases—as many as 20%—remain undiagnosed.^2^ Despite substantial progress made in Guinea since implementing the DOTS strategy, including a drastic reduction in mortality linked to this disease,^1 2^ the proportion of people with TB going undetected and therefore untreated means TB transmission may continue for long periods of time, while inappropriate use of medications may increase the risk of drug-resistant forms of TB,^3 4^ whose management is an ongoing challenge for the NTP.

Routine TB screening in high-risk groups enhances TB detection and progresses towards end TB goals.^5^ Active case finding through pharmacies in high TB burden settings may be feasible and high yield, as in many settings, up to 50% of people with symptomatic TB seek care in the private sector, including pharmacies.^4 6 7^ Pharmacy-based case finding efforts have also been shown to be effective through interventions supported by TB REACH across 15 countries. For example, pharmacy-based efforts in Vietnam led to a 32% increase in TB notifications, and in Nigeria, over 4000 additional people with TB were detected.^8^ However, undetected TB significantly contributes to the TB epidemic due to ongoing transmission.^9^,^11^ Early detection and treatment not only benefit individuals but also prevents further transmission within the community.

Integrating TB screening into pharmacy-based care can be a crucial first step in interrupting the chain of transmission. In this study, we implemented and evaluated an intensified TB case-finding strategy targeting customers who bought cough suppressants from pharmacies or drug outlets in Guinea, with the goal of increasing active TB detection and reducing the number of people with undiagnosed TB.

## Methods

### Study design and setting

We implemented and evaluated a strategy to improve early detection and treatment of TB disease in Matoto, Conakry, Guinea—the largest urban subprefecture in the Conakry urban area with a population of about 700 000—by engaging pharmacies and drug outlets. Pharmacies in this context refer to licensed establishments where qualified pharmacists dispense medications, while drug outlets include informal settings where medications are sold without pharmaceutical supervision. For this study, we strategically selected 25 of the most frequented pharmacies out of the 358 located in Conakry.^12^ An equal number of drug outlets were chosen based on their high customer volume, although the total number of such outlets in the city is not formally documented. This selection process aimed to maximise the likelihood of identifying individuals with TB-related symptoms from a broad cross-section of the population.

We trained and incentivised pharmacists and outlet owners to refer customers who bought cough suppressants and had at least one TB-related symptom (prolonged cough, fever, night sweats or weight loss) to community health workers (CHWs) for sputum collection and TB testing. Pharmacists and outlet owners were provided an information note to support patient identification and recruitment and were actively engaged throughout the study to ensure adherence with study protocols. Between November 2019 and June 2020, we consecutively enrolled eligible individuals who visited these sites, contacted the CHWs and verbally consented to participate in the study. The CHWs collected sputum samples from the participants either at a designated ventilated area at the pharmacy or outlet or at the nearest TB diagnosis and treatment centre (CDT). We performed acid-fast bacilli (AFB) smear microscopy for all participants and Xpert MTB/RIF for those with a previous history of TB disease. Data were registered on an open data kit (ODK) app on mobile devices. Test results were communicated to the participants by phone, if possible. Participants testing negative received education about TB prevention measures, while those testing positive were initiated on standard treatment according to the NTP guidelines. The entire process, from data registration to treatment initiation, was supervised by the research team to ensure accuracy, consistency and adherence to the study protocol. Online supplemental appendix figure 1 shows a flowchart of the intervention.

### Data collection and management

We used CHWs with extensive experience in data collection to record and manage participant information. Android phones equipped with the ODK application were configured and made available at each pharmacy, drug outlet, and CDT and used by the CHWs to collect the following sociodemographic and clinical variables: location identified (pharmacy or outlet), location where specimen provided (pharmacy, outlet, CDT), age, sex, TB symptoms (cough, fever, weight loss, night sweats), duration of symptoms, cohabitation with someone with TB disease and overall contact time, sputum sample characteristics, smear microscopy results and Xpert MTB/RIF results. We put a checklist in place, and we periodically analysed the data to check their quality and prevent and correct any errors. Trained members of the study team were responsible for entering and reporting data.

### Data analysis

We described the characteristics of the included population using number (%) for categorical data and median (IQR) for continuous data. We compared characteristics among those diagnosed with TB versus those not diagnosed using χ^2^ tests of independence, t-test and Wilcoxon rank-sum, as appropriate. We calculated the adjusted odds ratio (aOR) for TB diagnosis using multivariable logistic regression and adjusting for an a priori selected set of factors: age, sex, cough, fever, weight loss, night sweats, duration of symptoms, type of pharmacy and living with someone with TB for at least 5 days. We also evaluated how these characteristics were associated with visiting a pharmacy versus a drug outlet. We analysed the time-to-diagnosis from symptom onset of patients visiting pharmacies versus drug outlets according to the Kaplan-Meier method, and we used log-rank tests to compare survival curves. We used a Cox proportional hazards regression model—adjusted for the above factors—to determine factors associated with time to TB diagnosis from symptom onset. We considered p values less than 0.05 as statistically significant.

To estimate the costs of the intensified screening strategy from the healthcare system perspective in 2020 US$, we employed micro-costing, that is, a bottom-up approach. We converted all costs to US$ using purchasing power parity or exchange rates, as appropriate, when necessary. Unit costs were categorised as administrative (training, vehicles, fuel and telephony) or related to screening and treatment (pharmacist participation, sputum transport, patient evaluation, AFB smear, Xpert MTB/RIF and treatment). We estimated unit costs for each activity by identifying component costs and using various methods to estimate costs of each component. Personnel time was valuated using time estimation questionnaires and personnel time spent on specific activities. Capital expenditures, such as vehicles and Xpert MTB/RIF machines, were annuitised at 3% over a 5-year useful life. Lab overhead costs associated with smear and Xpert were estimated based on a previous study in Benin.^13^ Costs of treatment included those associated with medication, follow-up visits and monitoring tests. Costs of pharmacist participation were based on fixed costs paid per month to participating pharmacies and drug outlets. Training costs were prorated assuming it would be required annually.

We determined the total cost for each activity by multiplying the unit cost by the number of occurrences. We estimated the overall cost of the intervention by summing up the total costs for all activities. Additionally, we computed the cost per person referred and the cost per person diagnosed and treated for TB disease by dividing the total cost of the intervention by the respective numbers of people referred and number of people diagnosed and treated for TB.

### Patient and public involvement

Patients and the public were not involved in the design, execution, interpretation or dissemination of the study and its results.

## Results

We recruited 916 individuals from 50 pharmacies and drug outlets in Matoto, Conakry, Guinea; characteristics of the overall population and stratified by location (pharmacy vs drug outlet) are reported in table 1. We enrolled 457 (50%) participants from pharmacies and 459 (50%) from drug outlets. The median age of the participants was 31 years (IQR: 21–48), and 495 (54%) were male. The most prevalent symptoms were cough and fever, reported by 815 (89%) and 795 (87%) participants, respectively, which were more common among participants visiting drug outlets (p<0.001). The median duration of these symptoms was 14 days (IQR: 9–18). Only 134 (15%) participants reported living with someone with TB disease for at least 5 days. With respect to samples collected, most (745; 81%) were collected at the health facility; overall, 471 (51%) samples were salivary, 372 (41%) were mucopurulent, and 62 (6.8%) were muco-salivary.

**Table 1:** Characteristics of patients overall and by type of store where identified

Characteristic	N	Overall, n=916	Pharmacy, n=457	Drug outlet, n=459	P value*
Age	916	31 (21, 48)	30 (21, 47)	32 (22, 48)	0.6
Sex	916				0.7
Feminine		421 (46%)	213 (47%)	208 (45%)	
Male		495 (54%)	244 (53%)	251 (55%)	
Cough	916	815 (89%)	390 (85%)	425 (93%)	<0.001*
Weight loss	916	476 (52%)	221 (48%)	255 (56%)	0.035*
Fever	916	795 (87%)	344 (75%)	451 (98%)	<0.001*
Duration of symptoms (d)	916	14 (9, 18)	14 (12, 23)	14 (7, 14)	<0.001*
Previous TB exposure at home	916	139 (15%)	65 (14%)	74 (16%)	0.5
Duration of TB exposure (d)	134	5.0 (3.0, 6.0)	6.0 (3.5, 7.0)	4.0 (3.0, 6.0)	0.019
Location where sputum sample provided	916				<0.001*
CDT		745 (81%)	319 (70%)	426 (93%)	
Pharmacy or drug outlet		171 (19%)	138 (30%)	33 (7.2%)	
Sputum characteristics	916				<0.001*
Mucopurulent		372 (41%)	164 (36%)	208 (45%)	
Mucus		3 (0.3%)	0 (0%)	3 (0.7%)	
Salivary mucus		62 (6.8%)	62 (14%)	0 (0%)	
Purulent		5 (0.5%)	2 (0.4%)	3 (0.7%)	
Salivary		471 (51%)	228 (50%)	243 (53%)	
Sanguinolent		3 (0.3%)	1 (0.2%)	2 (0.4%)	
AFB smear microscopy result	916				0.018*
Negative		790 (86%)	407 (89%)	383 (83%)	
Positive		126 (14%)	50 (11%)	76 (17%)	
Xpert MTB/RIF result	11				0.5
Detected		3 (27%)	3 (38%)	0 (0%)	
Not detected		8 (73%)	5 (62%)	3 (100%)	
Rifampicin resistance on Xpert	6				
Not determined		5 (83%)	5 (83%)	0 (NA%)	
Invalid		1 (17%)	1 (17%)	0 (NA%)	

Data presented: median (IQR) or n (%).

* Statistical tests performed: Wilcoxon rank-sum test; χ2 test of independence; Fisher’s exact test.

AFB, acid-fast bacilli; CDT, Centre for the Diagnosis and Treatment of Tuberculosis; TB, tuberculosis .

We identified 126 (14%) people with smear-positive TB among the 916 participants who were referred and tested with AFB smear microscopy, with a larger fraction of participants testing positive at drug outlets compared with pharmacies (17% vs 11%; p=0.018). We performed Xpert MTB/RIF only for participants with a history of TB, that is, 11 patients, and detected TB in 3 (27%) of them; we did not detect any resistance to rifampicin. As shown in table 2, we found smear positivity was significantly associated with age (p=0.036), sex (p=0.006), self-reported cough (p<0.001), fever (p<0.001), weight loss (p<0.001), symptom duration (p<0.001) and cohabitation with TB patients (p=0.002). Sputum collection location (p=0.014) and appearance (p<0.001) were also positively correlated.

**Table 2:** Characteristics of patients according to microscopy results

Characteristic	N	Overall, n=916	Negative, n=790	Positive, n=126	P value*
Age	916	31 (21, 48)	32 (21, 49)	28 (20, 40)	0.036*
Sex	916				0.006*
Feminine		421 (46%)	378 (48%)	43 (34%)	
Male		495 (54%)	412 (52%)	83 (66%)	
Cough	916	815 (89%)	691 (87%)	124 (98%)	<0.001*
Fever	916	795 (87%)	670 (85%)	125 (99%)	<0.001*
Weight loss	916	476 (52%)	362 (46%)	114 (90%)	<0.001
Duration of symptoms (d)	916	14 (9, 18)	14 (9, 18)	14 (12, 17)	0.14
Previous TB exposure at home	916	139 (15%)	108 (14%)	31 (25%)	0.002*
Duration of TB exposure (d)	134	5.0 (3.0, 6.0)	5.0 (3.5, 6.0)	5.0 (2.5, 6.0)	0.5
Location where sputum sample provided	916				0.014*
CDT		745 (81%)	632 (80%)	113 (90%)	
Pharmacy or drug outlet		171 (19%)	158 (20%)	13 (10%)	
Sputum characteristics	916				<0.001*
Mucopurulent		372 (41%)	289 (37%)	83 (66%)	
Mucus		3 (0.3%)	3 (0.4%)	0 (0%)	
Salivary mucus		62 (6.8%)	57 (7.2%)	5 (4.0%)	
Purulent		5 (0.5%)	4 (0.5%)	1 (0.8%)	
Salivary		471 (51%)	434 (55%)	37 (29%)	
Sanguinolent		3 (0.3%)	3 (0.4%)	0 (0%)	
Xpert MTB/RIF result	11				0.2
Detected		3 (27%)	1 (12%)	2 (67%)	
Not detected		8 (73%)	7 (88%)	1 (33%)	
Rifampicin resistance on Xpert	6				0.2
Not determined		5 (83%)	5 (100%)	0 (0%)	
Invalid		1 (17%)	0 (0%)	1 (100%)	
Location identified	916				0.018*
Pharmacy		457 (50%)	407 (52%)	50 (40%)	
Drug outlet		459 (50%)	383 (48%)	76 (60%)	

Data presented: median (IQR) or n (%). Statistical tests performed: Wilcoxon rank-sum test; χ2 test of independence; Fisher’s exact test.

* p<0.05.

CDT, Centre for the Diagnosis and Treatment of Tuberculosis; TB, tuberculosis.

**Figure 1:**
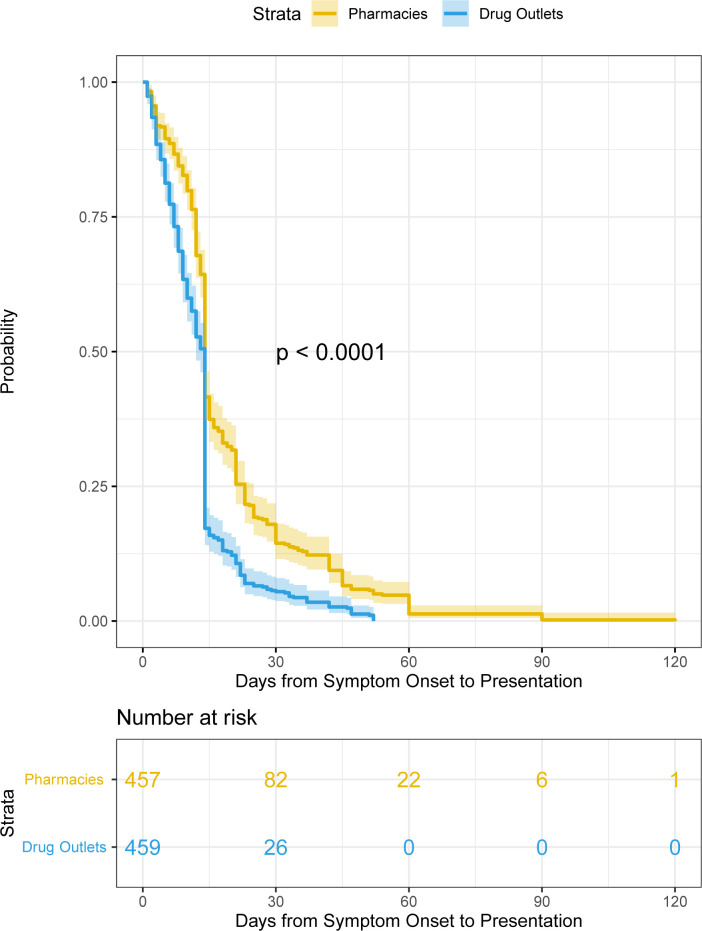
Comparison of time from symptom onset to screening for patients visiting pharmacies versus drug outlet pharmacies.

In multivariable analysis, increasing age (aOR=0.98; 95% CI 0.97 to 0.99 per year) significantly reduced the odds, while male sex (aOR=1.56; 95% CI 1.02 to 2.41), self-reported cough (aOR=7.90; 95% CI 2.36 to 49.1) and weight loss (aOR=11.3; 95% CI 6.27 to 22.4) significantly increased the odds of smear positivity (table 3). We found significant differences between participants visiting pharmacies versus drug outlets (table 3) only for those with self-reported fever in multivariable analysis (aOR=20.3; 95% CI 9.79 to 49.3). We found the time from symptom onset to presentation was significantly faster (shorter time) among those identified at drug outlets as opposed to pharmacies (mean time visiting outlets or 12.9 days and mean time visiting pharmacies of 19.5 days; adjusted HR=1.73; 95% CI 1.50 to 1.99) (table 3, figure 1).

**Table 3:** Multivariable analysis of characteristics associated with smear microscopy result, type of pharmacy frequented and time from symptoms to testing

Characteristic	Outcome					
	Microscopy result		Type of pharmacy		Time to test	
	OR (95% CI)	P value	OR (95% CI)	P value	HR (95% CI)	P value
Age (per year)	0.98 (0.97, 1.00)	0.012*	1 (1.00, 1.01)	0.4	0.99 (0.99, 1.00)	0.632
Sex						
Feminine	1 (reference)		1 (reference)		1 (reference)	
Male	1.56 (1.02, 2.41)	0.041*	0.98 (0.74, 1.30)	> 0.9	0.96 (0.84, 1.09)	0.506
Cough						
No	1 (reference)		1 (reference)		1 (reference)	
Yes	7.9 (2.36, 49.1)	0.005*	1.06 (0.62, 1.81)	0.8	0.91 (0.73, 1.14)	0.426
Fever						
No	1 (reference)		1 (reference)		1 (reference)	
Yes	10.9 (2.27, 196)	0.020*	20.3 (9.79, 49.3)	<0.001*	1.17 (0.86, 0.94)	0.161
Weight loss						
No	1 (reference)		1 (reference)		1 (reference)	
Yes	11.3 (6.27, 22.4)	<0.001*	1.07 (0.78, 1.46)	0.7	0.79 (0.69, 0.91)	0.0008*
Duration of symptoms (per day)	1 (1.00, 1.00)	> 0.9	1 (1.00, 1.00)	0.12	N/A	N/A
Live with a contact						
No	1 (reference)		1 (reference)		1 (reference)	
Yes	1.01 (0.61, 1.63)	> 0.9	1.01 (0.68, 1.52)	> 0.9	0.99 (0.82, 1.20)	0.925
Location identified						
Pharmacy	1 (reference)		N/A	N/A	1 (reference)	
Drug outlet	1.28 (0.84, 1.95)	0.3	N/A	N/A	1.73 (1.50, 1.99)	<0.001*
Results of microscopy						
Negative	N/A	N/A	1 (reference)		N/A	N/A
Positive	N/A	N/A	1.19 (0.78, 1.81)	0.4	N/A	N/A

* p<0.05

The total cost of the intervention was US$29 919, comprising US$6252 (21%) in administrative costs and US$23 667 (79%) in TB screening and treatment costs (table 4). The costliest components were pharmacist participation (US$8166 (US$27.22 per pharmacist per month)) and TB disease treatment ($11 462), which accounted for 66% of the total cost. The cost per person referred for sputum testing was US$32.66. The cost per person diagnosed and treated for smear-positive TB disease was US$237.45.

**Table 4:** Costs of the intervention

Cost	Number of occurrences	Cost per patient (2020 US$)	Total cost (2020 US$)
Administrative costs			
Space, materials, travel, personnel for training	–	–	US$2195
Bikes for sputum transport	–	–	US$3875
Fuel for bikes	–	–	US$312
Phone lines	–	–	US$870
TB screening and treatment costs			
Pharmacist participation	–	–	US$8166
Sputum transport from pharmacy	171	US$1.67	US$286
Patient evaluation after referral	916	US$0.93	US$852
Sputum smear microscopy	916	US$1.88	US$1722
Xpert MTB/RIF	11	US$16.28	US$179
TB disease treatment	126	US$90.97	US$11 462

TB, tuberculosis.

## Discussion

In this study, we demonstrated that intensified case finding for TB in pharmacies and drug outlets in the municipality of Matoto, Conakry, Guinea is a high-yield strategy to enhance TB diagnosis and treatment access with a modest cost. We found that approximately one in seven people referred for AFB smear microscopy from pharmacies and outlets had smear-positive TB, with males and those with cough and unintended weight loss with the highest odds of TB. Moreover, we found participants in the study who accessed drug outlets did so sooner from the time of symptom onset than those who accessed pharmacies, pointing to drug outlets as an important point of early intervention.

This is a success story of a Private-Public Mix TB care intervention. It is highly relevant to capitalise on diagnostic opportunities in pharmaceutical establishments. It is estimated that 3.6 million (36%) people living with TB disease globally go undiagnosed each year,^2^ and the three countries representing the largest diagnostic gap (India, Nigeria and Indonesia) have large community pharmacy sectors, providing an opportunity to detect and treat TB disease.

People often resort to self-medication using drugs purchased from the drug market or medicinal plants or seek care from traditional health specialists for their health problems. Approximately half of participants enrolled in this study were referred from drug outlets (50%), which may reflect the lower cost of drugs and/or the uncontrolled installation of drug outlets in Guinea, as speculated by others.^14^,^16^ Despite the Guinean government’s efforts to curb the illegal sale of medicines, including the ratification of the Council of Europe’s Convention on counterfeit medical products,^17^ this illicit trade persists and continues to pose a significant threat to timely TB diagnosis.

Clinically, cough (90%) and fever (88%) were the most frequent symptoms among participants with suspected TB, with a median duration of these symptoms of 14 days. These symptoms are common with endemic respiratory diseases in Guinea, and their persistence can motivate self-medication to relieve symptoms. Said *et al*^18^ showed the most common symptoms of TB to be cough (99.6%), weight loss (96%), night sweats (94%) and fever (93%). In an African context, repeated respiratory infections make coughing a common symptom, hence self-medication, for which pharmacies and drug outlets are preferred places for this practice.^19 20^

In our study, half (50%) of the samples collected for smear microscopy were salivary, followed by mucopurulent (43%). This contrasts with the samples collected in Uganda (2009–2011) and Brazil (2013–2015) in the study by Acuña-Villaorduña *et al*,^21^ which were mostly purulent and/or mucopurulent, that is, 59% and 96%, respectively. Sputum quality may influence TB diagnosis via smear microscopy; however, a systematic review^22^ found mixed evidence regarding the relationship between sputum quality and diagnostic performance. Collecting sputum specimens poses a challenge due to patient nonresponse to appointments. It sometimes requires home-visiting activities to collect samples and perform follow-up with household contacts if they test positive for TB. Therefore, the involvement of CHWs is essential to reach most of the patients who are identified through pharmacies and drug outlets.

Smear-positive TB was detected in nearly one in seven participants (14%). These findings could be explained by sociocultural and economic factors in Guinean communities; patients from underprivileged social strata usually turn to self-medication and traditional healers; these observations have been confirmed in Cameroon^18^ and Burkina Faso^23^ unlike high-income countries.^24 25^ In a study of an integrated, comprehensive referral and TB screening intervention in India by Daftary *et al*,^26^ a similar proportion of people referred for TB screening from pharmacies was found (15%), which was equivalent to increasing the rate of TB diagnosis 25-fold when compared with the pre-intervention period. Similarly, Sharma *et al*^27^ identified 871 people with TB over a 9-month period (a 3.2-fold increase in case notification) with the help of 330 pharmacists who were sensitised to the possibility of TB among customers buying medicines in India. The difference in the study population and design could explain these results; indeed, ours is a study on people seeking cough relief at pharmacies, while Daftary^26^ and Sharma^27^ involved people identified and diagnosed according to national recommendations. The expansion of the TB screening strategy to the pharmacy and drug outlet level in Guinea will necessitate a carefully planned initiative, guided by a thorough evaluation of the existing strategy’s strengths and weaknesses. Furthermore, it must be intricately structured to capitalise on the valuable insights garnered from previous experiences, seamlessly incorporating them into the framework to amplify effectiveness and extend outreach.

We noted a significant difference between pharmacies and drug outlets regarding time from symptom onset to presentation at each of these facilities. In a previous review of time from symptom onset to presentation at health facilities, the average symptom duration was 36 days,^28^ which is longer than the average duration seen in our study of 19 days for pharmacies and 13 days for drug outlets. These differences are consistent with these informal drug outlets being a point of first contact for many with symptoms. However, outside of this study, the management of drug outlets by people who generally have no medical training and therefore may not be able to guide patients to an appropriate health facility may result in significant diagnostic delays, as was observed in Ethiopia.^29^ Factors such as age, male gender, cough and weight loss were significantly associated with smear positivity in our study as often reported in the literature.^23 30^

Overall, the cost of these intensified case finding activities were modest at US$32.66 per person referred for sputum testing and US$237.45 per person diagnosed and treated. These values are comparatively lower to active case finding activities in Cambodia (US$373, 2015) and Tajikistan (US$343, 2015) targeted to elderly individuals in rural communities and different clinical settings, respectively.^31^ These costs also fall well below the average US$864 (2010) per person diagnosed with TB in the first TB REACH funding cycle and may be considered highly cost-efficient when compared with other case finding activities.^32^ These lower costs may be due to use of inexpensive smear microscopy as opposed to Xpert MTB/RIF, which is more expensive but can detect people with smear-negative TB. Use of Xpert among all participants initially smear-negative would increase the cost per person referred from US$32.66 to US$46.51. Even in the unlikely scenario, no additional person was diagnosed and treated for TB; the cost per person diagnosed and treated would increase from US$237.45 to US$338.10—still well below costs of other active case finding interventions.

Our study is subject to several notable limitations. First, as this was an operational research project, we did not collect, nor expect pharmacists or outlet owners to collect, data on the total number of people presenting with symptoms who purchased medication. Thus, the proportion of people who potentially could have benefited from TB disease testing who were not tested is unknown. It is also possible this group might have been systematically different from the participants who were referred to the study team (eg, milder symptoms), and so our results should be interpreted in this context. Second, the constrained availability of the Xpert MTB/RIF test within our facilities resulted in its usage being confined to participants with a TB history, potentially leading to the oversight of cases involving smear-negative or rifampicin-resistant TB. Third, the study’s limited 1-year timeframe hindered an evaluation of the intervention’s influence on critical patient outcomes, including cure rates, relapses or mortality. Fourth, the challenge of accumulating data on additional medications purchased from pharmacies was attributed to the substantial work burden on pharmacists, impeding the completion of our designated forms.

Given the positive outcomes of this study, a next logical step would be to evaluate the TB case finding strategy in other regions of Guinea. To effectively plan for scale-up, it will be essential to map and analyse the catchment areas of pharmacies and drug outlets across the country and understand health seeking behaviour of the population. This would ensure a comprehensive understanding of where the strategy can be most impactful and would help identify any regional variations in healthcare access that could influence the outcomes. Further research should focus on these aspects to guide a more tailored and effective nationwide implementation.

## Conclusion

In summary, this study demonstrated that intensified TB case finding through pharmacies and drug outlets in Matoto, Conakry, is both feasible and effective in enhancing TB detection in a context where self-medication is prevalent and TB remains underdiagnosed. By strategically engaging both formal pharmacies and informal drug outlets, we were able to identify a significant number of people with TB that might have otherwise gone undetected. The findings highlight the critical role of the pharmaceutical sector in identifying individuals with TB symptoms and facilitating their access to diagnosis and treatment. Given the success of this intervention, expanding this approach on a larger scale could significantly contribute to reducing the TB burden in Guinea. However, further research and careful planning will be necessary to optimise the implementation of this strategy across diverse settings and to ensure its sustainability and cost-effectiveness.

## Supplementary material

10.1136/bmjresp-2024-002334 online supplemental file 1

## Data Availability

Data are available upon reasonable request.
