# Supplementary figures and images for: Tuberculosis screening among cough suppressant buyers in pharmacies and drug outlets in Guinea: a cross-sectional study
Source: BMJ Open Respir Res. 2024 Dec 31;11(1):e002334. doi: 10.1136/bmjresp-2024-002334 (PMC13059888; doi:10.1136/bmjresp-2024-002334)

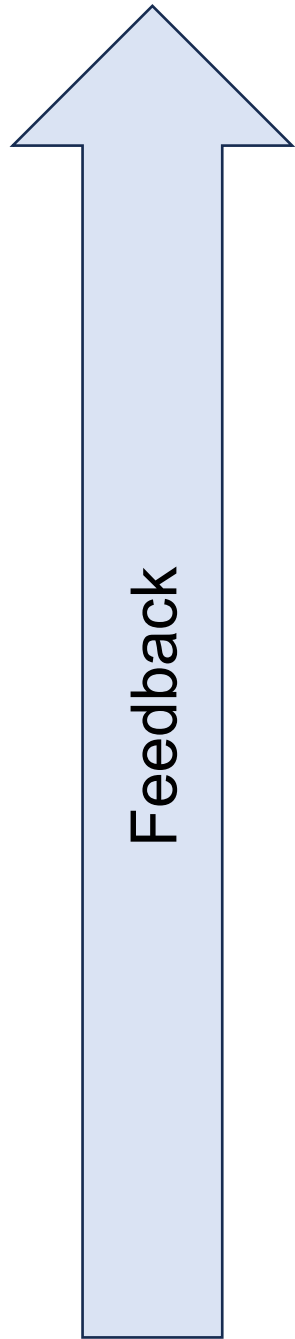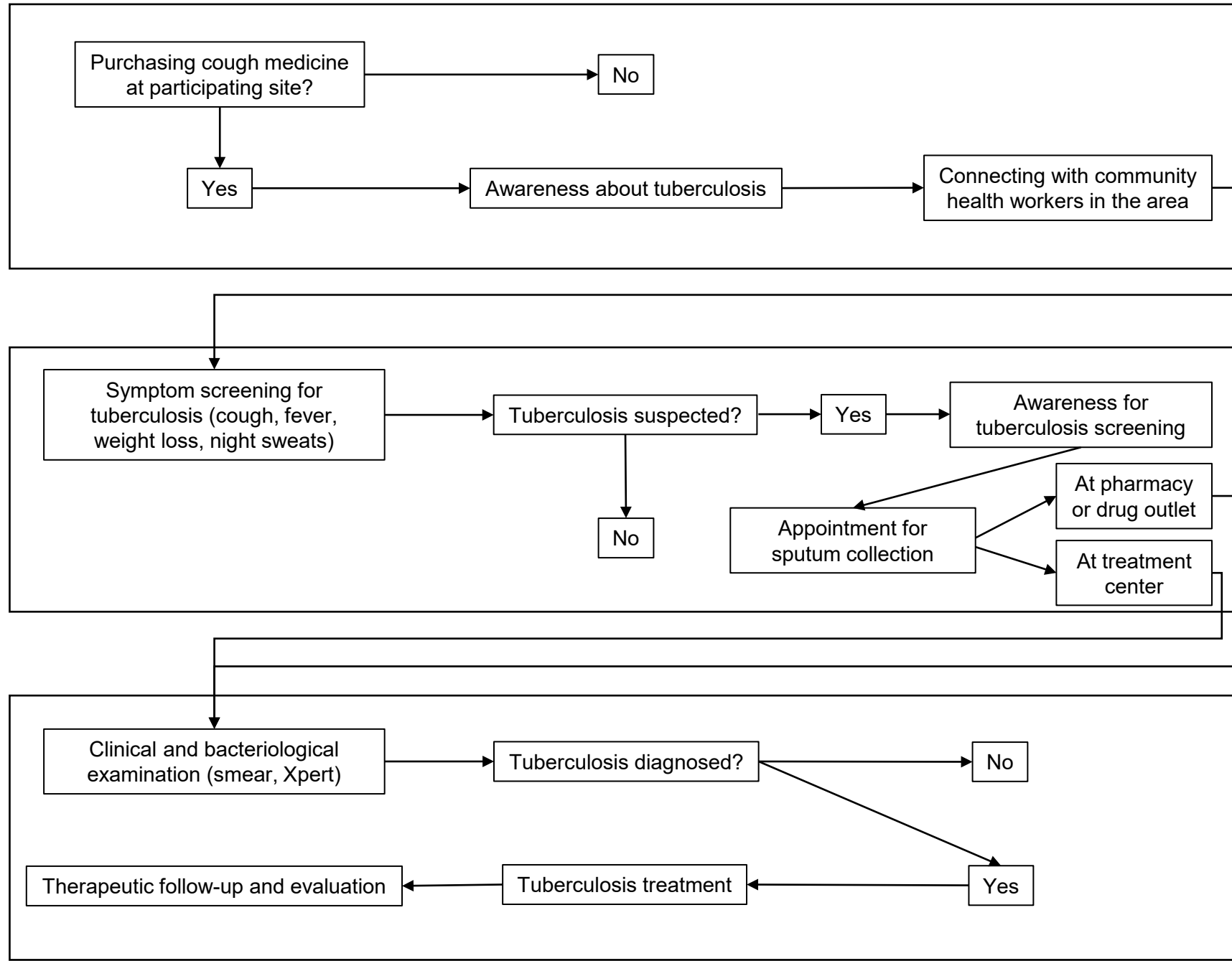

Pharmacy / Drug Outlet

Community Level

Tuberculosis Diagnosis and Treatment Centre

Supplement: online supplemental file 1 [file bmjresp-11-1-s001.pdf]
